# Incidence of retinal vein occlusion with long-term exposure to ambient air pollution

**DOI:** 10.1371/journal.pone.0222895

**Published:** 2019-09-24

**Authors:** Han-Wei Zhang, Chao-Wen Lin, Victor C. Kok, Chun-Hung Tseng, Yuan-Pei Lin, Tsai-Chung Li, Fung-Chang Sung, Chi Pang Wen, Chao A. Hsiung, Chung Y. Hsu

**Affiliations:** 1 PhD Program for Aging, China Medical University, Taichung, Taiwan; 2 Institute of Electrical Control Engineering, Department of Electrical and Computer Engineering, National Chiao Tung University, Hsinchu, Taiwan; 3 Institute of Population Health Sciences, National Health Research Institutes, Zhunan, Taiwan; 4 Department of Ophthalmology, National Taiwan University Hospital, Taipei, Taiwan; 5 Disease Informatics Research Group, Asia University Taiwan, Taichung, Taiwan; 6 Department of Internal Medicine, Kuang Tien General Hospital, Taichung, Taiwan; 7 Department of Neurology, China Medical University Hospital, and School of Medicine, China Medical University, Taichung, Taiwan; 8 Graduate Institute of Biostatistics, College of Public Health, China Medical University, Taichung, Taiwan; 9 Department of Healthcare Administration, College of Health Science, Asia University, Taichung, Taiwan; 10 Graduate Institute of Clinical Medical Science and School of Medicine, College of Medicine, China Medical University, Taichung, Taiwan; 11 Graduate Institute of Biomedical Science, China Medical University, Taichung, Taiwan; University of Mississippi Medical Center, UNITED STATES

## Abstract

This study aimed to investigate whether long-term exposure to airborne hydrocarbons, including volatile organic compounds, increases the risk of developing retinal vein occlusion (RVO) among the population of Taiwan. A retrospective cohort study involving 855,297 people was conducted. Cox proportional hazards regression analysis fitted the multiple pollutant models for two targeted pollutants, including total hydrocarbons (THC), nonmethane hydrocarbons (NMHC) were used, and the risk of RVO was estimated. The chi-squared test and one-way analysis of variance were used to test differences in demographics and comorbidity distribution among tertiles of the targeted pollutants. Before controlling for multiple pollutants, hazard ratios for the overall population were 19.88 (95% CI: 17.56–22.50) at 0.51-ppm increases in THC and 4.33 (95% CI: 3.97–4.73) at 0.27-ppm increases in NMHC. The highest adjusted hazard ratios for different multiple pollutant models of each targeted pollutant were statistically significant (all *p* values were ≤0.05) for all patients at 29.67 (95% CI: 25.57–34.42) for THC and 16.24 (95% CI: 14.14–18.65) for NMHC. Our findings suggest that long-term exposure to THC and NMHC contribute to RVO development.

## Introduction

Retinal vein occlusion (RVO), the second most common retinal vascular disease, is characterized by painless vision loss [1]. Patients with RVO may have a reduced quality of life and functional activities of daily living [2]. RVO occurs as a result of retinal vein thrombosis due to external compression of an atherosclerotic artery or increased blood viscosity, much like a stroke. If the retinal vein is blocked, it cannot drain blood from the retina, leading to retinal hemorrhage, vascular tortuosity, cotton wool spots, and optic disc edema. Increased vascular pressure leads to fluid leakage and cystoid macular edema, a vision-threatening complication. Eyes with capillary nonperfusion may develop ocular neovascularization, which also carries a risk of vision loss. RVO can be classified into branch retinal vein occlusion (BRVO), hemiretinal vein occlusion, and central retinal vein occlusion (CRVO) depending on the obstruction site.

The worldwide prevalence of BRVO is approximately 0.4%, whereas that of CRVO is approximately 0.08% [3]. Overall, 16 million people are affected in one or both eyes, with equal distribution between men and women, and a higher risk with older age. BRVO is more common than CRVO. The development of RVO correlates with typical risk factors for atherosclerosis [4], but other processes may play roles, including inflammation, compression, thrombophilic conditions, or vasospasm [5].

Extensive research has been performed to investigate the adverse health effects of air pollution. These air particles are associated with prothrombotic states, endothelial dysfunction, atherosclerosis progression, and increased systemic oxidative stress [6–7]. Air pollution is an established trigger of cardiovascular events [8]. Moreover, studies have reported that extended exposure to air pollution matter is a key predictor of cardiopulmonary disease mortality [9–10]. Individuals with RVO have a higher risk of developing ischemic stroke and hemorrhagic stroke [11]. Because RVO and ischemic stroke share some common attributes in pathogenesis and risk factors, air pollution could be an important, modifiable risk factor for RVO.

Established in 1996, the National Health Institute Research Database (NHIRD) in Taiwan is a Longitudinal Health Insurance Database that stores electronic health records for health beneficiaries. NHIRD, which contains health care data of 22.96 million people (99% of the population in Taiwan) under a universal health insurance program [12], constitutes real-world practice outcomes or evidence that is increasingly recognized for its significance and clinical impact beyond the results drawn from multicenter clinical trials that establish the “evidence” for which to develop clinical practice guidelines [13]. NHIRD provides high-quality health care data with integrity and reliability for exploring real-world evidence based on big health data analytics.

Taiwan’s sustainable energy policy, a new policy developed by the Bureau of Energy, Ministry of Economic Affairs [14], possesses a framework that aims to boost electricity generation from low carbon emission natural gas plants and renovate coal-fired and thermal power stations relying on diesel engines. These endeavors will lead to future long-term exposure to airborne hydrocarbons because the combustion of fuels produces air pollutants in the form of gases, volatile organic compounds (VOCs), and particulate matter. Systemic and ocular risk factors associated with RVO are well documented; however, little is known about the factors that can trigger an RVO event. Therefore, the present study aimed to assess the adverse impact of air pollution of total hydrocarbons (THC) and nonmethane hydrocarbons (NMHC) on human health by focusing on RVO. Data from NHIRD and government environmental databases were used to determine whether long-term exposure to hydrocarbons in ambient air increased the risk of RVO among the population of Taiwan.

## Materials and methods

### Data sources

The data used in the present study were obtained from the Longitudinal Health Insurance Database 2000 (LHID2000) within NHIRD, which claims data for 1 million random samples from 1996 to 2013. The observation period was set as 2000–2013 to enhance the NHIRD data reliability. The Environment Resource Dataset [15] was publicly available from open government data. The Environmental Protection Administration of Taiwan assessed the levels of ambient pollutants measured by 76 monitoring stations across Taiwan from 1993 to 2013. The Research Ethics Committee of China Medical University and Hospital in Taiwan approved the study, with the certificate number CMUH-104-REC2-115-CR3.

### Study design and study population

**Fig 1:**
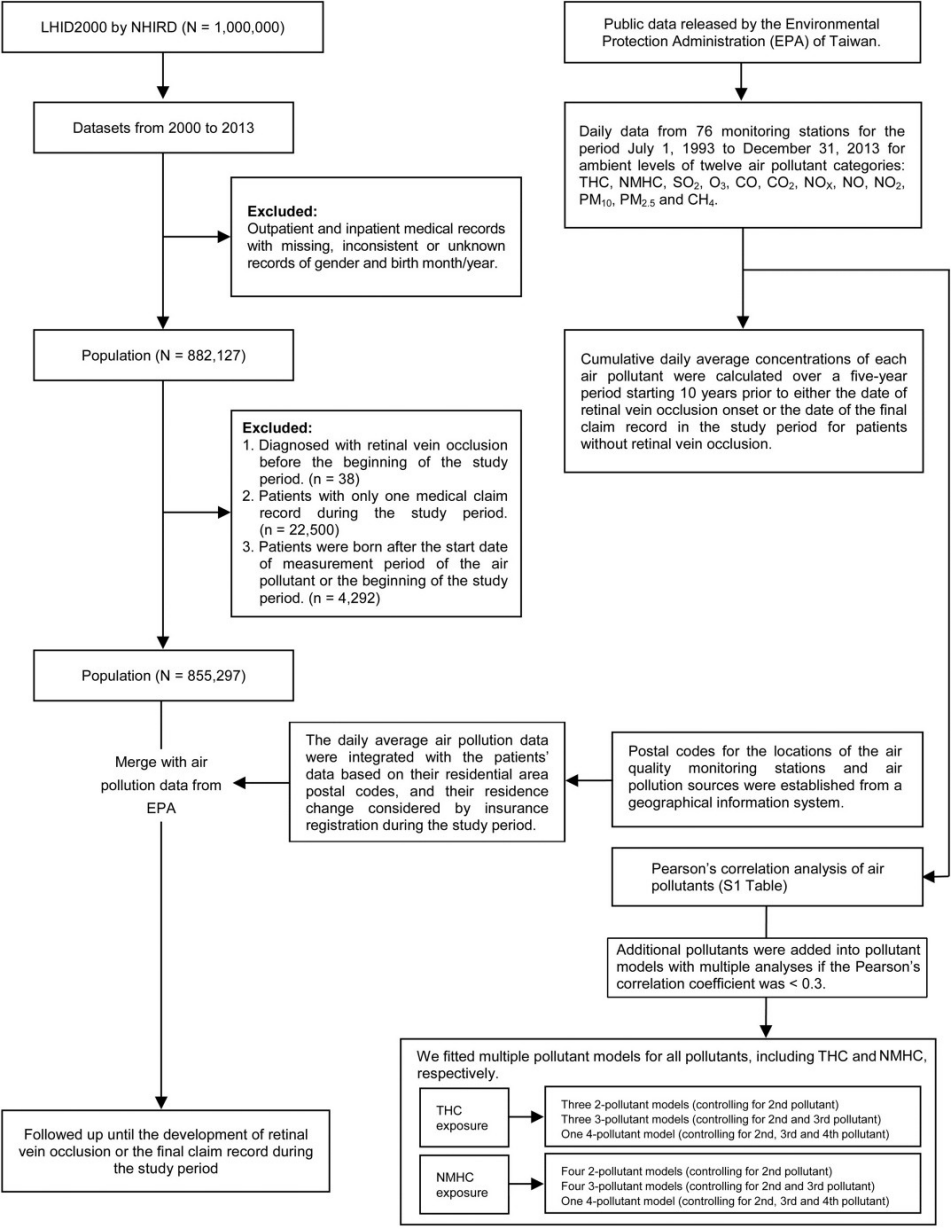
Summary of study flow.

We used a cohort design and set the study period from January 1, 2000 to December 31, 2013. Fig 1 summarizes the selection of study population. Of the 1 million patients in the LHID2000 database, we excluded patients with missing or unknown records for gender or birth year and month, individuals with a diagnosis of RVO before January 1, 2000 (n = 38), those with only one claim record during the study period (n = 22,500), and those who were born after the start date of the measurement period of the air pollutant or the beginning of the study period (n = 4,292). Ultimately, 855,297 patients were included in the present study.

### Outcome selection

From the included population, we identified those with a first-time diagnosis of RVO during the study period, based on International Classification of Diseases, Ninth Revision, Clinical Modification (ICD-9-CM) codes 362.35 and 362.36. Patients were considered to have RVO if they had visited an outpatient clinic ≥ 3 times and were diagnosed with RVO or had ever been hospitalized due to RVO. The earliest date of hospitalization or outpatient visit with the diagnosis was considered as the date of diagnosis and served as the newly diagnosed date of RVO for all subsequent analyses.

### Exposure measurement for the targeted pollutants

To examine the associations between newly diagnosed RVO with long term exposure to targeted air pollutants and consider the multiple pollutants effect by controlling other non-targeted pollutants over the exposure period, we calculated the concentrations of 12 pollutant categories monitored by the Environmental Protection Administration in Taiwan. The present study targeted THC and NMHC on the basis of their weak correlation (i.e., with correlation coefficients < 0.3) with 10 other monitored categories: sulfur dioxide (SO_2_), ozone (O_3_), carbon monoxide (CO), carbon dioxide (CO_2_), nitrogen oxides (NO_X_), nitrogen monoxide (NO), nitrogen dioxide (NO_2_), fine particulate matter < 10 μm in size (PM_10_), fine particulate matter < 2.5 μm in size (PM_2.5_), and methane (CH_4_) (S1 Table). Non-targeted pollutants were included in subsequent multiple pollutant analyses. From July 1, 1993 to December 31, 2013, daily air-quality data were collected by 76 monitoring stations and maintained by the Environmental Protection Administration [16]. We recorded the locations with air pollutants to establish an integrated geographic information system. Using this system, each patient was linked to the appropriate monitoring region via postal code and the residence change considered by insurance registration during the study period. A patient’s long-term exposure to each pollutant category was defined as the daily average cumulative concentration during the measurement period, calculated for a 5-year period starting either 10 years before RVO onset or 10 years before the date of the final claim record in the study period for patients without RVO. Therefore, long-term exposure (*LEAP*) for each pollutant category (*i* = THC, NMHC, SO_2_, O_3_, CO, CO_2_, NO_X_, NO, NO_2_, PM_10_, PM_2.5_ and CH_4_) for a patient living in the region served by air quality monitoring station *j* was calculated as follows:

$$LEAP_{ij}\text{=}\frac{\sum_{t=m}^{n}AP_{ijt}}{d}$$

where *AP*_i_ is the level of ambient air pollution for pollutant category; *i*, *m* is the start date of the measurement period (either 10 years before the date of RVO onset or 10 years before the date of the final claim record in the study period for patients without RVO); *n* is the end date of the measurement period (either 5 years before the date of RVO onset or 5 years before the date of the final claim record in the study period for patients without RVO); and *d* is the number of days in the measurement period.

### Comorbidities

Information on the patients' comorbid conditions was obtained from LHID2000 based on ICD-9-CM codes. Comorbidities examined in the analysis included coronary artery disease (410–414), chronic kidney disease (580–587), chronic obstructive pulmonary disease (491, 492, 494, and 496), asthma (493), arrhythmia (427, 785.0, and 785.1), cerebrovascular diseases (430–438), hypertension (401–405), diabetes mellitus (249, 250, 648.0, and 648.8), hyperlipidemia (272), smoking-related diagnosis (305.1, 491.0, 491.2, 492.8, 496, 523.6, 649.0, 989.84, and V15.82), morbid obesity (278, 646.1, 649.1, 649.2, V45.86, V65.3, and V77.8), glaucoma (364.22, 365, 366.31, and 377.14), hypercoagulable state (289.81, 325, 415.1, 451.1, 451.2, 451.81, 451.83, 451.84, 451.89, 451.9, 452, 453.0–453.4, and 453.8–453.9), and retinal arteriolar disorder (362.31–362.34). We identified and defined these conditions according to the diagnostic history collected from at least three outpatient visits or a single hospital admission prior to the newly diagnosed date of RVO.

### Statistical analysis

The chi-squared test (for categorical variables) and one-way analysis of variance (for continuous variables) were used to test the differences in demographics and comorbidity distribution among tertiles of the targeted pollutant concentrations. The incidences of newly diagnosed RVO were calculated per 10,000 person-years. For the analyses of the time to the diagnosis of RVO, each individual’s observation time was censored at the date of the final-claim record during the study period. Cox proportional hazards regression was used to examine the risk of RVO associated with each targeted pollutant category, expressed as hazard ratios (HRs) with 95% confidence intervals (CIs). Multivariate Cox proportional hazards regression, which takes into account potential confounders, the possible link of air pollutants, was used to examine the effects of multiple pollutants, controlling for other pollutants based on the selection of weak correlations with other air pollutants (i.e., correlation coefficients between each both of air pollutants were lower than 0.3; S1 Table). To avoid potential collinearity problems, we did not include pollutants with high correlations in the same regression model. The effect of each targeted pollutant on the risk of newly diagnosed RVO was estimated as the adjusted HRs for the change in per interquartile range (IQR) of 0.51 ppm for THC and of 0.27 ppm for NMHC over the follow-up period.

Several studies showed the association of air pollution exposure with cardiovascular disorders in conjunction with the synergistic effect of temperature. Temperature was added in particulate air pollution model to control the effect of weather conditions on air pollution and stroke mortality [17] because both cold and hot temperatures were associated with the increased risk of stroke mortality [18]. Elevated mortality from cardiovascular diseases has been shown to be related to extreme temperature; the increase and decrease in ambient temperature had a relationship with the cardiovascular mortality [19–20]. Therefore, to control the effect of weather conditions on air pollution and RVO, ambient temperature would be one of the adjusting factors in the pollutant models. In the present study, the multiple pollutant models were fitted for the two targeted pollutants and estimated their independent effects by adjusting for age, gender, ambient temperature, and the unbalanced distribution of comorbidities (*p* ≤ 0.05), controlling for pollutants with weak correlations in the models. To test for potential dose–response relationships, the data from concentration of each targeted pollutant category were divided into three levels by the tertiles, and adjusted HRs with 95% CIs were calculated again.

Stratified analyses were performed to determine whether the effects of the pollutant categories were different between males and females. Kaplan–Meier analysis plots were used to determine the probability of people remaining without RVO, and the log-rank test was used to examine the difference among tertiles of concentrations of pollutant categories. The analyses were performed using CareStore X1 Studio Research Platform and the Statistical Product and Service Solutions (SPSS; Version 22). All statistical tests were two-sided, and *p* values ≤ 0.05 were considered statistically significant.

## Results

### Characteristics of the study population

Demographic data and comorbid states among tertiles of targeted pollutant categories are presented in Tables 1 and 2, with T1 being the lowest level and T3 the highest. The mean age of analysis initiation was 30.5 ± 20.8, 31.9 ± 19.6, and 37.2 ± 20.3 years at T1, T2, and T3 levels, respectively, for THC. For NMHC, the mean ages were 38.3 ± 18.5, 27.4 ± 21.9, and 34.7 ± 18.9 years at T1, T2, and T3 levels, respectively. Assessing 14 comorbidities among tertiles of targeted pollutant categories revealed imbalances for these comorbidities, which were adjusted in subsequent analyses.

**Table 1:** Characteristics of the study population among tertiles of total hydrocarbons (THC) exposure.

Characteristics	Tertiles of average daily THC^*a*^, n (%)			*P* value^*b*^	Total (*n* = 837,917)
	T1 (lowest) (*n* = 279,308)	T2 (*n* = 280,917)	T3 (highest) (*n* = 277,692)		
**Retinal vein occlusion**	115 (0.04)	254 (0.1)	1,138 (0.4)	<0.001	1,507 (0.2)
**Age**, years				<0.001	
Mean ± SD	30.5 ± 20.8	31.9 ± 19.6	37.2 ± 20.3		33.2 ± 20.4
**Gender**				<0.001	
Male	137,707 (49.3)	144,907 (51.6)	148,915 (53.6)		431,529 (51.6)
**Coronary artery disease**	30,030 (10.8)	28,301 (10.1)	31,596 (11.4)	<0.001	89,927 (10.9)
**Chronic kidney disease**	14,224 (5.1)	14,362 (5.1)	20,036 (7.2)	<0.001	48,622 (5.9)
**Chronic obstructive pulmonary disease**	22,986 (8.2)	22,235 (7.9)	24,279 (8.7)	<0.001	69,500 (8.4)
**Asthma**	28,878 (10.3)	24,097 (8.6)	19,981 (7.2)	<0.001	72,956 (8.8)
**Arrhythmia**	33,141 (11.9)	29,563 (10.5)	27,282 (9.8)	<0.001	89,986 (10.8)
**Cerebrovascular diseases**	20,449 (7.3)	20,374 (7.3)	26,860 (9.7)	<0.001	67,683 (8.2)
**Hypertension**	50,865 (18.2)	47,434 (16.9)	41,124 (14.8)	<0.001	139,423 (16.7)
**Diabetes mellitus**	34,712 (12.4)	33,632 (12.0)	36,014 (13.0)	<0.001	104,358 (12.6)
**Hyperlipidemia**	51,681 (18.5)	50,194 (17.9)	41,231 (14.8)	<0.001	143,106 (17.0)
**Smoking-related diagnosis**	12,543 (4.5)	13,670 (4.9)	15,374 (5.5)	<0.001	41,587 (5.0)
**Morbid obesity**	2,413 (0.9)	2,354 (0.8)	1,964 (0.7)	<0.001	6,731 (0.8)
**Glaucoma**	7,064 (2.5)	7,707 (2.7)	7,088 (2.6)	<0.001	21,859 (2.6)
**Hypercoagulable state**	1,452 (0.5)	1,550 (0.6)	2,427 (0.9)	<0.001	5,429 (0.7)
**Retinal arteriolar disorder**	91 (0.03)	94 (0.03)	117 (0.04)	0.116	302 (0.04)

Note: SD, standard deviation.

^*a*^Tertile values in ppm were as follows: T1 level: < 2.28, T2 level: ≥ 2.28 and < 2.40, T3 level: ≥ 2.40.

^*b*^The chi-squared test or one-way analysis of variance among tertiles of total hydrocarbons.

**Table 2:** Characteristics of the study population among tertiles of nonmethane hydrocarbons (NMHC) exposure.

Characteristics	Tertiles of average daily NMHC^*a*^, n (%)			*P* value^*b*^	Total (n = 837,917)
	T1 (lowest) (n = 251,321)	T2 (n = 294,887)	T3 (highest) (n = 291,709)		
**Retinal vein occlusion**	383 (0.2)	351 (0.1)	773 (0.3)	<0.001	1,507 (0.2)
**Age**, years				<0.001	
Mean ± SD	38.3 ± 18.5	27.4 ± 21.9	34.7 ± 18.9		33.2 ± 20.4
**Gender**				<0.001	
Male	130,689 (52.0)	147,136 (49.9)	153,704 (52.7)		431,529 (51.6)
**Coronary artery disease**	34,868 (13.9)	26,674 (9.0)	28,385 (9.7)	<0.001	89,927 (10.9)
**Chronic kidney disease**	17,570 (7.0)	14,667 (5.0)	16,385 (5.6)	<0.001	48,622 (5.9)
**Chronic obstructive pulmonary disease**	26,852 (10.7)	21,555 (7.3)	21,093 (7.2)	<0.001	69,500 (8.4)
**Asthma**	21,274 (8.5)	31,047 (10.5)	20,635 (7.1)	<0.001	72,956 (8.8)
**Arrhythmia**	36,512 (14.5)	27,802 (9.4)	25,672 (8.8)	<0.001	89,986 (10.8)
**Cerebrovascular diseases**	26,274 (10.5)	19,592 (6.6)	21,817 (7.5)	<0.001	67,683 (8.2)
**Hypertension**	53,729 (21.4)	41,463 (14.1)	44,231 (15.2)	<0.001	139,423 (16.7)
**Diabetes mellitus**	39,624 (15.8)	30,669 (10.4)	34,065 (11.7)	<0.001	104,358 (12.6)
**Hyperlipidemia**	53,559 (21.3)	43,259 (14.7)	46,288 (15.9)	<0.001	143,106 (17.0)
**Smoking-related diagnosis**	16,145 (6.4)	12,068 (4.1)	13,374 (4.6)	<0.001	41,587 (5.0)
**Morbid obesity**	2,147 (0.9)	2,102 (0.7)	2,482 (0.9)	<0.001	6,731 (0.8)
**Glaucoma**	7,127 (2.8)	7,438 (2.5)	7,294 (2.5)	<0.001	21,859 (2.6)
**Hypercoagulable state**	1,921 (0.8)	1,533 (0.5)	1,975 (0.7)	<0.001	5,429 (0.7)
**Retinal arteriolar disorder**	107 (0.04)	88 (0.03)	107 (0.04)	0.046	302 (0.04)

Note: SD, standard deviation.

^*a*^Tertile values in ppm were as follows: T1 level: < 0.29, T2 level: ≥ 0.29 and < 0.36, T3 level: ≥ 0.36.

^*b*^The chi-squared test or one-way analysis of variance among tertiles of non-methane hydrocarbons.

### Associations between retinal vein occlusion and pollutant categories

Table 3 shows the single and multiple pollutant models for per IQR of 0.51-ppm increases in THC. Before controlling for other pollutants, the results determined that newly diagnosed RVO had a positive association with the daily average concentration over the 5-year period for THC, with an adjusted HR of 19.88 (95% CI: 17.56–22.50, *p* < 0.001), indicating that a 0.51-ppm increase in THC increased the likelihood of having RVO by 1888%. The adjusted HRs (95% CIs) were different between males and females (17.75 [14.96–21.06] and 22.82 [19.08–27.28], respectively, all *p* < 0.001). Table 3 also details the changes in adjusted HRs for different multiple pollutant models. Controlling for second and third pollutants (SO_2_ and NO_2_) produced the highest adjusted HRs of 29.67 (25.57–34.42), 23.02 (18.77–28.24), and 40.49 (32.56–50.35) for all patients, males and females, respectively.

**Table 3:** Hazard ratios for RVO risk associated with long-term THC exposure at a 0.51-ppm increment, controlled for the concentrations of other air pollutants.

Population	Controlling pollutant^*a*^	Adjusted HR^*b*^ (95% CI)
Total (n = 855,297)	-	19.88 (17.56,22.50)^‡^
	SO_2_	16.30 (14.42,18.43)^‡^
	O_3_	10.11 (8.91,11.48)^‡^
	NO_2_	19.56 (16.94,22.58)^‡^
	SO_2_, O_3_	7.14 (6.38,7.98)^‡^
	SO_2_, NO_2_	29.67 (25.57,34.42)^‡^
	O_3_, NO_2_	11.92 (10.06,14.12)^‡^
	SO_2_, O_3_, NO_2_	23.63 (19.93,28.01)^‡^
Male (n = 441,290)	-	17.75 (14.96,21.06)^‡^
	SO_2_	14.76 (12.43,17.52)^‡^
	O_3_	9.55 (8.02,11.38)^‡^
	NO_2_	16.61 (13.62,20.26)^‡^
	SO_2_, O_3_	6.68 (5.71,7.83)^‡^
	SO_2_, NO_2_	23.02 (18.77,28.24)^‡^
	O_3_, NO_2_	10.28 (8.14,12.98)^‡^
	SO_2_, O_3_, NO_2_	17.84 (14.12,22.54)^‡^
Female (n = 414,007)	-	22.82 (19.08,27.28)^‡^
	SO_2_	18.55 (15.58,22.09)^‡^
	O_3_	10.85 (9.02,13.04)^‡^
	NO_2_	24.08 (19.55,29.65)^‡^
	SO_2_, O_3_	7.81 (6.67,9.15)^‡^
	SO_2_, NO_2_	40.49 (32.56,50.35)^‡^
	O_3_, NO_2_	14.40 (11.25,18.42)^‡^
	SO_2_, O_3_, NO_2_	33.51 (26.00,43.19)^‡^

Note: HR, hazard ratio; 95% CI, 95% confidence interval; THC, total hydrocarbons; SO_2_, sulfur dioxide; O_3_, ozone; NO_2_, nitrogen dioxide.

^*a*^Additional pollutants were added into THC models for the multiple analysis only when the Pearson’s correlation coefficient was < 0.3.

^*b*^Cox regression models were adjusted for age, gender, coronary artery disease, chronic kidney disease, chronic obstructive pulmonary disease, asthma, arrhythmia, cerebrovascular diseases, hypertension, diabetes mellitus, hyperlipidemia, smoking-related diagnose, morbid obesity, glaucoma, hypercoagulable state, and ambient temperature, controlling pollutants (weak correlation with THC).

^‡^*p* < 0.001.

Table 4 shows the single and multiple pollutant models for per IQR of 0.27-ppm increases in NMHC. Before controlling for multiple pollutants, the adjusted HRs for the overall population, males and females were 4.33 (3.97–4.73), 4.28 (3.80–4.83), and 4.45 (3.92–5.06), respectively. The multiple pollutant models controlling PM_2.5_ had the highest adjusted HRs of 16.24 (14.14–18.65), 18.70 (15.18–23.05), and 15.14 (12.56–18.24), respectively.

**Table 4:** Hazard ratios for RVO risk associated with long-term exposure to NMHC, at a 0.27-ppm increment, controlled for the concentrations of other air pollutants.

Population	Controlling pollutant^*a*^	Adjusted HR^*b*^ (95% CI)
Total (n = 855,297)	-	4.33 (3.97,4.73)^‡^
	SO_2_	3.65 (3.36,3.98)^‡^
	O_3_	2.69 (2.50,2.90)^‡^
	NO_2_	3.47 (3.06,3.94)^‡^
	PM_2.5_	16.24 (14.14,18.65)^‡^
	SO_2_, O_3_	2.31 (2.14,2.49)^‡^
	SO_2_, NO_2_	4.17 (3.67,4.74)^‡^
	O_3_, NO_2_	1.42 (1.27,1.58)^‡^
	O_3_, PM_2.5_	7.79 (6.81,8.92)^‡^
	SO_2_, O_3_, NO_2_	1.91 (1.69,2.17)^‡^
Male (n = 441,290)	-	4.28 (3.80,4.83)^‡^
	SO_2_	3.60 (3.21,4.05)^‡^
	O_3_	2.67 (2.41,2.96)^‡^
	NO_2_	3.41 (2.87,4.05)^‡^
	PM_2.5_	18.70 (15.18,23.05)^‡^
	SO_2_, O_3_	2.31 (2.07,2.56)^‡^
	SO_2_, NO_2_	3.98 (3.34,4.73)^‡^
	O_3_, NO_2_	1.45 (1.25,1.69)^‡^
	O_3_, PM_2.5_	9.69 (7.90,11.88)^‡^
	SO_2_, O_3_, NO_2_	1.89 (1.60,2.23)^‡^
Female (n = 414,007)	-	4.45 (3.92,5.06)^‡^
	SO_2_	3.79 (3.35,4.29)^‡^
	O_3_	2.76 (2.47,3.08)^‡^
	NO_2_	3.64 (3.01,4.40)^‡^
	PM_2.5_	15.14 (12.56,18.24)^‡^
	SO_2_, O_3_	2.34 (2.08,2.62)^‡^
	SO_2_, NO_2_	4.47 (3.70,5.40)^‡^
	O_3_, NO_2_	1.40 (1.19,1.65)^‡^
	O_3_, PM_2.5_	6.66 (5.56,7.99)^‡^
	SO_2_, O_3_, NO_2_	1.97 (1.64,2.37)^‡^

Note: HR, hazard ratio; 95% CI, 95% confidence interval; NMHC, nonmethane hydrocarbons; SO_2_, sulfur dioxide; O_3_, ozone; NO_2_, nitrogen dioxide; PM_2.5_, fine particulate matter < 2.5 μm in size.

^*a*^Additional pollutants were added into NMHC models for the multiple analysis only when the Pearson’s correlation coefficient was < 0.3.

^*b*^Cox regression models were adjusted for age, gender, coronary artery disease, chronic kidney disease, chronic obstructive pulmonary disease, asthma, arrhythmia, cerebrovascular diseases, hypertension, diabetes mellitus, hyperlipidemia, smoking-related diagnose, morbid obesity, glaucoma, hypercoagulable state, retinal arteriolar disorder, and ambient temperature, controlled pollutants (weak correlation with NMHC).

^‡^*p* < 0.001.

### The dose–response relationship between air pollutants and retinal vein occlusion

Table 5 highlights the Cox proportional hazards regression analysis of the two targeted pollutant categories divided into tertiles. The lowest tertile was used as the reference in each case, and the estimated HRs were adjusted for age, gender, ambient temperature, and disparities in the prevalence of comorbidities, which is consistent with the results obtained from the earlier multivariate analyses.

**Table 5:** The dose–response association between air pollutants and RVO risk, by gender.

Pollutant category	Tertiles of average daily pollutant^*a*^	Population	RVO	PY	Adjusted HR^*b*^ (95% CI)
THC	T1 (lowest)	Total (n = 855,297)	115	3,715,993	1 (Ref)
	T2		254	3,644,148	2.33 (1.87,2.91)^‡^
	T3 (highest)		1,138	2,995,396	13.52 (11.06,16.53)^‡^
	T1 (lowest)	Male (n = 441,290)	67	1,818,182	1 (Ref)
	T2		124	1,868,926	1.84 (1.37,2.48)^‡^
	T3 (highest)		587	1,597,860	10.80 (8.27,14.09)^‡^
	T1 (lowest)	Female (n = 414,007)	48	1,897,812	1 (Ref)
	T2		130	1,775,222	3.02 (2.17,4.20)^‡^
	T3 (highest)		551	1,397,536	17.31 (12.74,23.53)^‡^
NMHC	T1 (lowest)	Total (n = 855,297)	383	3,174,514	1 (Ref)
	T2		351	3,782,958	1.09 (0.94,1.26)
	T3 (highest)		773	3,398,065	2.59 (2.25,2.97)^‡^
	T1 (lowest)	Male (n = 441,290)	193	1,635,739	1 (Ref)
	T2		175	1,875,529	1.16 (0.95,1.43)
	T3 (highest)		410	1,773,699	2.63 (2.17,3.19)^‡^
	T1 (lowest)	Female (n = 414,007)	190	1,538,774	1 (Ref)
	T2		176	1,907,429	1.02 (0.83,1.25)
	T3 (highest)		363	1,624,366	2.55 (2.09,3.11)^‡^

Note: RVO, retinal vein occlusion; PY, person-years; HR, hazard ratio; 95% CI, 95% confidence interval; THC, total hydrocarbons; NMHC, nonmethane hydrocarbons.

^*5*:*21 PMa*^Tertile values in ppm (THC, NMHC) were as follows:

THC (T1 level: < 2.28, T2 level: ≥ 2.28 and < 2.40, T3 level: ≥ 2.40); NMHC (T1 level: < 0.29, T2 level: ≥ 0.29 and < 0.36, T3 level: ≥ 0.36).

^*b*^Cox regression models were adjusted for age, gender, comorbidities, and ambient temperature.

*p < 0.05,

^‡^*p* < 0.001.

In those exposed to the highest tertile (T3) of THC for the overall population, the adjusted HR was 13.52 (11.06–16.53), indicating that those exposed to average daily levels of ≥ 2.40 ppm THC were 1252% more likely to have newly diagnosed RVO than those exposed to < 2.28 ppm (values corresponding with the tertiles are shown in the legend for Table 5). The adjusted HRs for T3 of THC were 10.80 (8.27–14.09) for males and 17.31 (12.74–23.53) for females.

For NMHC, the adjusted HRs for T3 exposure relative to T1 exposure for the overall population, males, and females were 2.59 (2.25–2.97), 2.63 (2.17–3.19), and 2.55 (2.09–3.11), respectively. When data on gender were stratified or merged for analysis, statistically significant correlations of adjusted HRs were measured for T1 compared with T2 and T3. The analysis revealed dose–response relations for the two targeted pollutants, irrespective of gender.

Cumulative incidence of RVO for the two targeted pollutants was assessed using the Kaplan–Meier method (S1 Fig), which demonstrated a clear trend of an increased risk of RVO as each targeted pollutant exposure increased. Statistically significant differences were found in RVO occurrence among tertiles of targeted pollutant categories (log-rank test, *p* < 0.001).

## Discussion

This population-based cohort study linked national insurance claims data to open government data to investigate the association between long-term exposure to selected air pollutants in Taiwan and the risk of developing RVO. To the best of our knowledge, these are the first results to show an adverse impact of VOCs on the risk of developing RVO in the ambient air in individuals who were exposed to three concentrations of average daily levels over a 5-year period. The adjusted HRs for newly diagnosed RVO among individuals exposed to the highest concentration level of THC exceeded 13-fold, compared with the lowest concentration level of THC.

The anthropogenic emission of VOCs, including different types of hydrocarbons, has a significant environmental impact on human health and atmospheric photochemistry [21]. Air pollutants, including CH_4_, are important atmospheric precursors of ozone (O_3_) with emission by industry and traffic-related air pollutants contributing to particulate matter (PM) pollution [22]. Studies have shown that THC and NMHC play a critical role in the photochemical production of O_3_ and organic aerosols [23]. O_3_ is considered the most toxic air pollutant and has various adverse health effects. A previous animal study showed that ozone exposure in mice demonstrated increased damage in the conjunctival goblet cells and corneal integrity and increased the production of inflammatory cytokines in the tears in a dose-dependent manner [24]. Cerebrovascular disease is also linked to increased levels of O_3_, and an association has been made between stroke and airborne PM [25]. High levels of O_3_ and PM, major pollutants listed in the Pollutants Standard Index in Taiwan [26], from industry and transportation vehicles are associated with retinal microvascular dysfunction and central nervous system diseases, including ischemic stroke [27].

Atherosclerosis, a chronic low-grade inflammatory condition, is a distinct type of arteriosclerosis caused by plaque buildup in the walls of the arteries. This buildup narrows the arteries, which can restrict blood flow, causing a heart attack or stroke. Both systemic and local inflammations are thought to play significant roles in RVO etiology. The systemic risk factors of RVO are independently associated with atherosclerosis. The pathological findings of atherosclerosis comprise monocyte-derived macrophages and T-lymphocytes (purely inflammatory lesions), causing central retinal vein compression and subsequent collapse, thereby impeding blood flow, which subsequently progresses to thrombus and clot formation, resulting in a vein occlusion [28].

Many inflammatory cytokines are prothrombogenic, and systemic inflammation may induce hypercoagulability. Interleukin-1 beta, tumor necrosis factor-alpha, and interleukin-6 upregulate tissue factor and activate the extrinsic coagulation cascade pathway. They also downregulate tissue-type plasminogen activators and disrupt fibrinolysis. Finally, they can cause systemic thrombotic adverse events [29].

Adar et al. [30] reported on the short-term effects of air pollution on the human retinal microvasculature. They were the first to associate air pollution exposure with arterial narrowing, which is a risk factor of hypertension, myocardial infarction, and cardiovascular mortality. Retinal blood vessels share similarities with the microvasculature of the heart and brain [31]. The retina possesses a blood-retinal barrier, which is analogous to the blood-brain barrier. Pathological changes in the retinal vasculature may be similarly reflected in the cerebral vessels [32].

Toxicology studies have shown that microvascular responses are associated with short-term exposure to peak levels of air pollutants [33]. Retinal vessel caliber is a predictor for cardiovascular diseases; retinal arterial narrowing is a marker for predicting hypertension, and venular widening is associated with inflammation and endothelial dysfunction. In terms of endothelial dysfunction, systemic inflammatory response may take time to affect the retinal vessels. Inflammatory responses may alter the endothelial activity and cause endothelial dysfunction, possibly resulting in retinal arteriolar narrowing, even several hours after exposure [34]. A study by Zhang et al. [35] found that decreased microvascular endothelial function was related to increased exposure to air pollution.

However, the present study has several limitations. First, it is a retrospective study; hence, potential biases resulting from unknown confounders associated with the adjustment of confounding factors may have affected the results. Moreover, we were not able to adjust for confounders, such as genetic information and relevant clinical variables (e.g., imaging results, physiologic levels), because the relevant information was not available in the NHIRD. Except for age and gender, we considered 14 risk factors as potential risk factors for RVO. Because of the paucity of data sources, the effects of other clinical factors, including the levels of total cholesterol, creatinine, serum lipid, blood pressure, plasma lipoprotein, plasma fibrinogen, and body mass index, were not assessed in the current study [4,36–39]. RVO is generally more common in elderly, hypertensive patients, and people with cardiovascular diseases. With the global ageing trend, the prevalence and burden of RVO are also likely to expand. More epidemiological studies on RVO incidence are still needed to better understand the disease burden of RVO [39]. Second, our exposure assessment relied on residences and accounted for each patient's registration of moving during the course of the study, which still does not completely reflect personal exposure. To protect patient privacy, the NHIRD does not provide patient addresses, workplaces, and types. Therefore, we used the participants' insurance registrations during the exposure period in the study to assign them residential districts according to postal codes, which could have led to exposure misclassification, resulting in attenuated study results.

## Conclusions

Our findings suggest that THC and NMHC are pathological agents contributing to RVO development. We also confirmed the diagnosis of comorbidities in NHIRD, strengthening the possible link of air pollutants, including THC and NMHC, with increased risk for developing RVO (S2 Fig). Our stratified analyses revealed that long-term exposure to each of these two targeted pollutants was significantly associated with the increased risk for developing RVO, with different pronounced effects in females and males.

## Supporting information

S1 Table Pearson’s correlation analysis for air pollutants at baseline from July 1, 1993 to December 31, 2013. (PDF)

S1 Fig Cumulative incidence of retinal vein occlusion for individuals among tertiles of pollutant categories: (A) THC and (B) NMHC. The tertile values, in ppm (THC, NMHC), were as follows: THC (T1 level: < 2.28, T2 level: ≥ 2.28 and < 2.40, T3 level: ≥ 2.40); NMHC (T1 level: < 0.29, T2 level: ≥ 0.29 and < 0.36, T3 level: ≥ 0.36)
(PDF)

S2 Fig Visual abstract for the study. (PDF)
