# Supplementary material for: Incidence of retinal vein occlusion with long-term exposure to ambient air pollution
Source: PLoS One. 2019 Sep 24;14(9):e0222895. doi: 10.1371/journal.pone.0222895 (PMC6759191; doi:10.1371/journal.pone.0222895)
Supplement: S1 Table — (PDF) [file pone.0222895.s001.pdf]

S1 Table. Pearson's correlation analysis for air pollutants at baseline from July 1, 1993 to December 31, 2013

	SO_2	O_3	CO	CO_2	NO_X	NO	NO_2	PM_{10}	$PM_{2.5}$	THC	NMHC	CH_4
SO_2	1	**−0.069**†	0.379†	0.306†	0.378†	**0.264**†	**0.007**†	0.384†	0.484†	**0.257**†	**0.294**†	**0.124**†
O_3		1	**−0.247**†	**−0.178**†	−0.346†	−0.399†	**−0.001**	**0.221**†	**0.268**†	**−0.103**†	**−0.184**†	**−0.010**†
CO			1	0.378†	0.911†	0.863†	0.750†	0.343†	0.383†	0.717†	0.850†	0.303†
CO_2				1	0.451†	0.342†	0.465†	0.425†	0.528†	0.434†	0.532†	**0.176**†
NO_X					1	0.935†	**0.010**†	0.351†	0.323†	0.686†	0.826†	**0.277**†
NO						1	**0.007**†	**0.180**†	**0.134**†	0.612†	0.779†	**0.203**†
NO_2							1	**0.007**†	0.519†	**0.006**†	**0.006**†	0.347†
PM_{10}								1	0.859†	0.334†	0.307†	**0.235**†
$PM_{2.5}$									1	0.363†	**0.284**†	0.321†
THC										1	0.828†	0.799†
NMHC											1	0.335†
CH_4												1

SO_2, sulfur dioxide; O_3, ozone; CO, carbon monoxide; CO_2, carbon dioxide; NO_X, nitrogen oxides; NO, nitrogen monoxide; NO_2, nitrogen dioxide; PM_{10}, fine particulate matter < 10 μm in size; $PM_{2.5}$, fine particulate matter < 2.5 μm in size; THC, total hydrocarbons; NMHC, nonmethane hydrocarbons; CH_4, methane.

†Correlation significant at the 0.01 level (two-tailed).

Correlation coefficient values of <0.3 denote a low strength of correlation, which qualified as the controlling pollutant in multiple-pollutant models of targeted pollutants.
