# Supplementary material for: Incidence of retinal vein occlusion with long-term exposure to ambient air pollution
Source: PLoS One. 2019 Sep 24;14(9):e0222895. doi: 10.1371/journal.pone.0222895 (PMC6759191; doi:10.1371/journal.pone.0222895)
Supplement: S1 Fig — Cumulative incidence of retinal vein occlusion for individuals among tertiles of pollutant categories: (A) THC and (B) NMHC. The tertile values, in ppm (THC, NMHC), were as follows: THC (T1 level: < 2.28, T2 level: ≥ 2.28 and < 2.40, T3 level: ≥ 2.40); NMHC (T1 level: < 0.29, T2 level: ≥ 0.29 and < 0.36, T3 level: ≥ 0.36) (PDF) [file pone.0222895.s002.pdf]

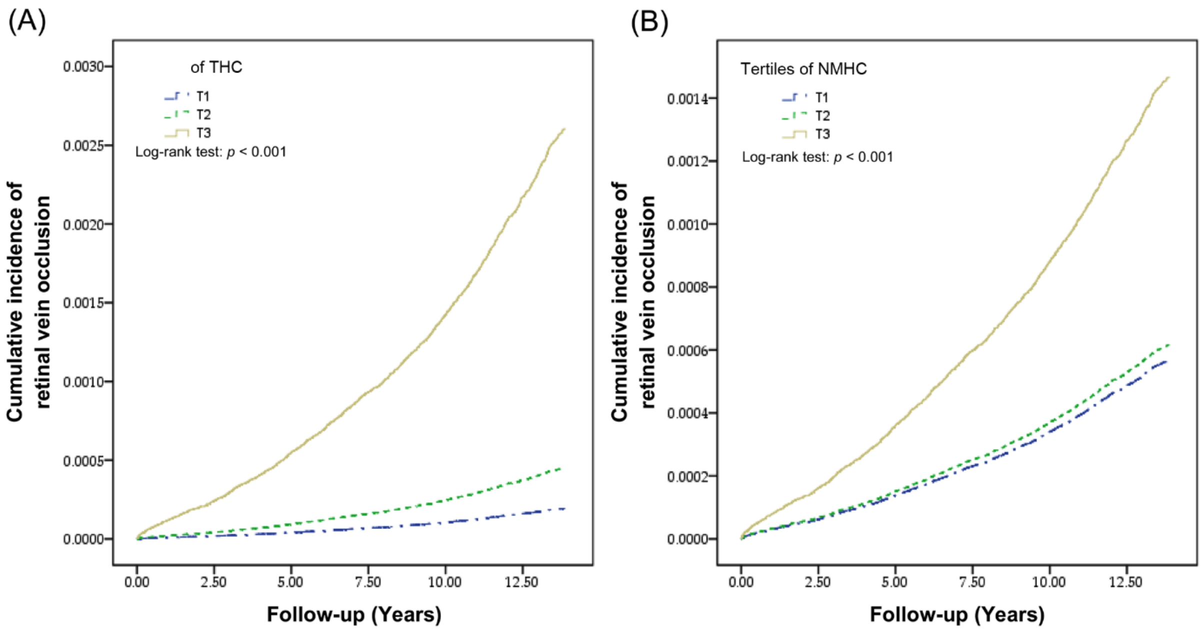

S1 Fig. Cumulative incidence of retinal vein occlusion for individuals among tertiles of pollutant categories: (A) THC and (B) NMHC

The tertile values, in ppm (THC, NMHC), were as follows: THC (T1 level: < 2.28, T2 level: ≥ 2.28 and < 2.40, T3 level: ≥ 2.40); NMHC (T1 level: < 0.29, T2 level: ≥ 0.29 and < 0.36, T3 level: ≥ 0.36).
