# Supplementary material for: Incidence of retinal vein occlusion with long-term exposure to ambient air pollution
Source: PLoS One. 2019 Sep 24;14(9):e0222895. doi: 10.1371/journal.pone.0222895 (PMC6759191; doi:10.1371/journal.pone.0222895)
Supplement: S2 Fig — (PDF) [file pone.0222895.s003.pdf]

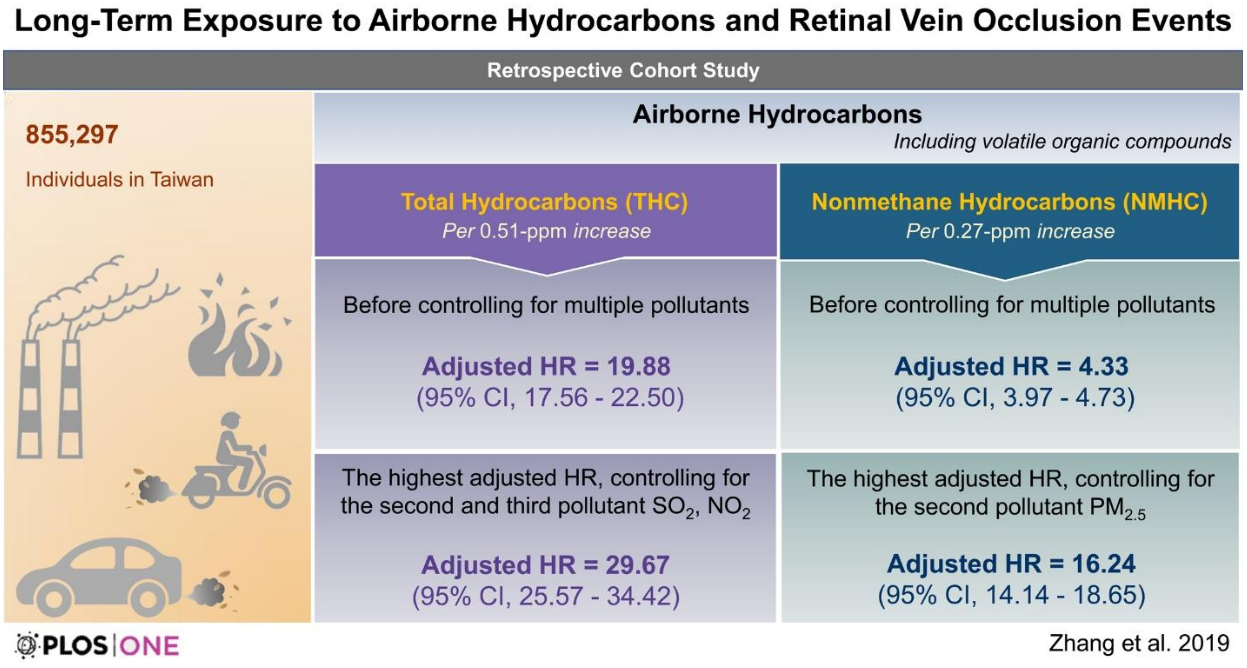

S2 Fig. Visual abstract for the study
